# Supplementary material for: Atomic visualization of a non-equilibrium sodiation pathway in copper sulfide
Source: Nat Commun. 2018 Mar 2;9:922. doi: 10.1038/s41467-018-03322-9 (PMC5834500; doi:10.1038/s41467-018-03322-9)
Supplement: Supplementary file 1 — Supplementary Information [file 41467_2018_3322_MOESM1_ESM.pdf]

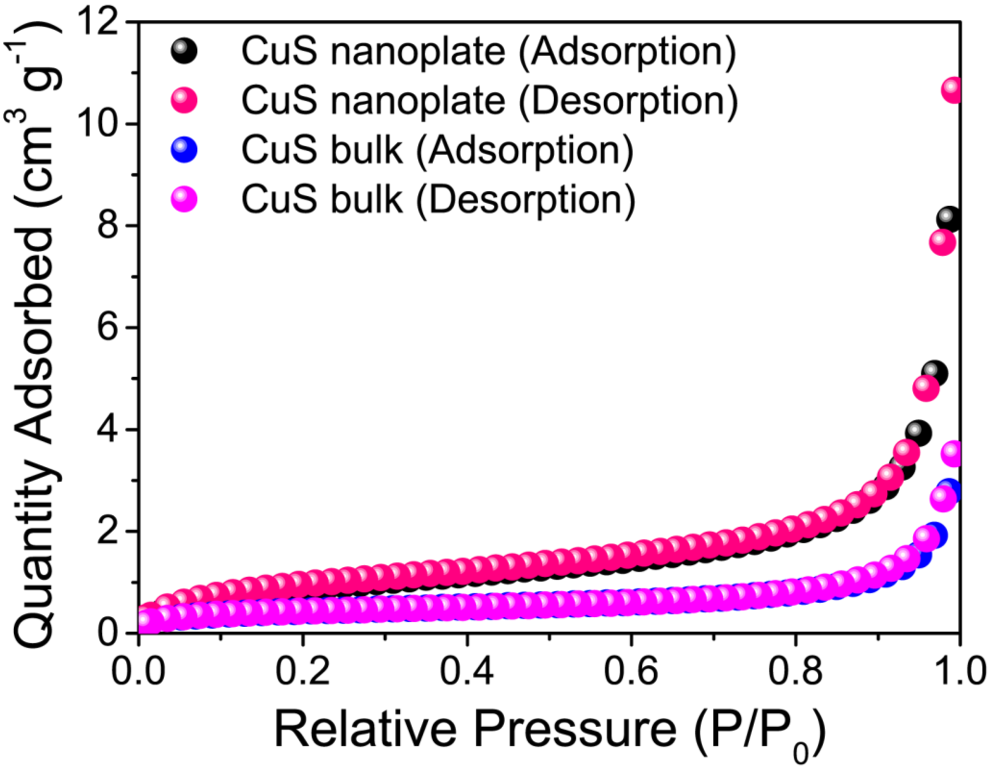

Supplementary Figure 1. Brunauer−Emmett−Teller (BET) measurement of CuS nanoplates and bulk. CuS nanoplates have ~2.26 times higher surface area than the CuS bulk. Both nanoplates and bulk have no pores since adsorption and desorption branches are almost same in relative pressure between 0.5 and 1.0.

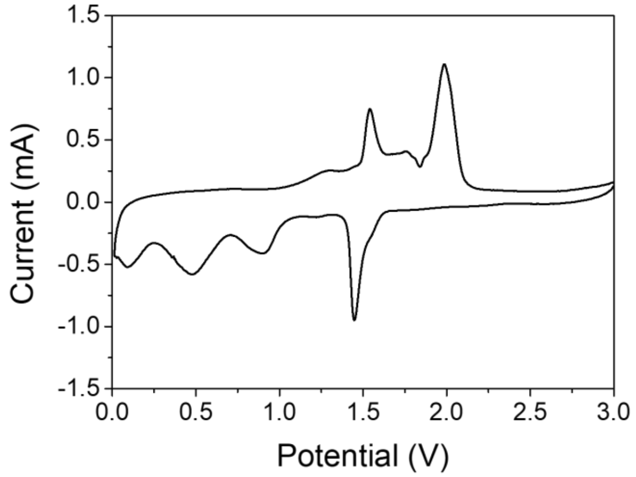

Supplementary Figure 2. Cyclic voltammetry profile of CuS after 150 cycles at the voltage scan rate of 0.2 mV/s. Cathodic peaks appear at 1.45 V, 0.9 V, 0.48 V and 0.09 V, which correspond to phase transitions of CuS to $Na(CuS)_4$, $Na(CuS)_4$ to $Na_7(Cu_6S_5)_2$, $Na_7(Cu_6S_5)_2$ to $Na_3(CuS)_4$, and $Na_3(CuS)_4$ to Na_2S/Cu. On the other hand, anodic peaks are observed at 1.99 V, 1.76 V, 1.54 V and 1.29 V. No noticeable peak is observed above 2.4 V and below 0.05 V; A capacity contribution from those voltage windows is negligible.

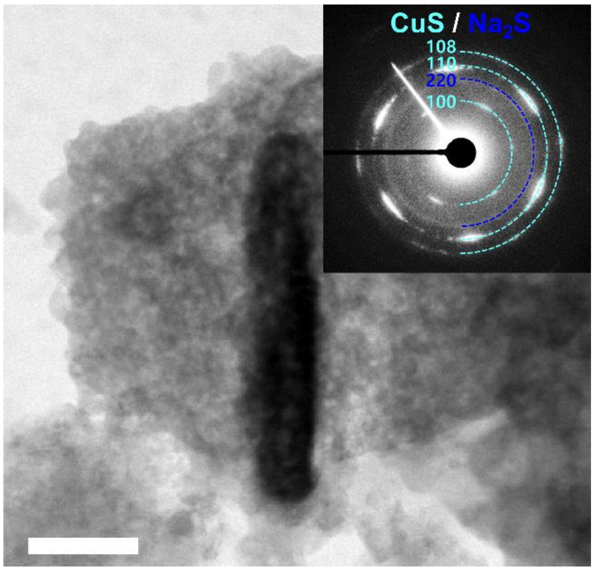

Supplementary Figure 3. Na_xCuS nanoplate after 20 charge/discharge cycles. Low-magnification TEM image of the nanoplate starting to disintegrate into smaller parts but still maintaining its original shape (scale bar, 100 nm). Note that the diffused diffraction spots in the selected area electron diffraction (SAED) pattern suggest the relative mis-orientation of individual grains fractured from the pristine CuS nanoplate.

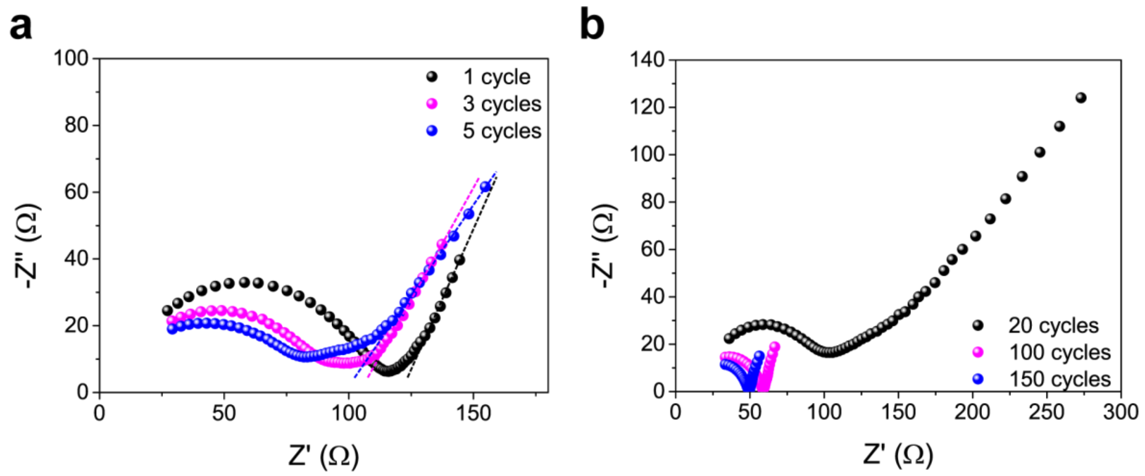

Supplementary Figure 4. Nyquist plots from electrochemical impedance spectroscopy (EIS) results upon cycling. (a) The EIS performed after 1st, 3rd and 5th and **(b)** after 20th, 100th and 150th discharges within the frequency range between 10 kHz and 0.1 Hz at amplitude of 10 mV. In the EIS, profiles in high-medium frequency are attributed to charge-transfer resistance (R_{ct}) while low frequency regions are associated with bulk diffusion of Na ions. For the initial 5 cycles, R_{ct} and the slope of low frequency regions decrease. The decrease of R_{ct} can be associated with the CuS nanoplate fracturing into nano-sized grains during cycles. A decline in the slope is related to the loss of Na mobility inside the CuS nanoplate because an exposed area is not enough for Na insertion and extraction. For the 20th, 100th, and 150th cycles, R_{ct} still decreases while the slope increases from 20 cycles to 100 cycles and almost retains; this means that a CuS nanoplate is further disintegrated into smaller size, while Na mobility is enhanced. The disintegration increases the exposed surface area of CuS, reduces its R_{ct}, and thus facilitates insertion and extraction of Na ions.

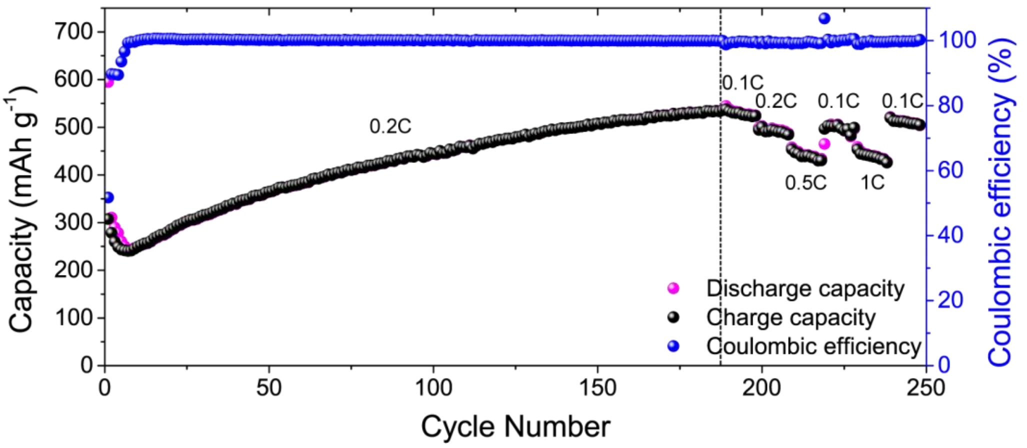

Supplementary Figure 5. Electrochemical performance of CuS nanoplates for >150 cycles. The cell is operated between 0.05 V and 2.4 V until ~ 190 cycles, followed by C-rate dependent capacity measurement between 0.05 V and 2.0 V.

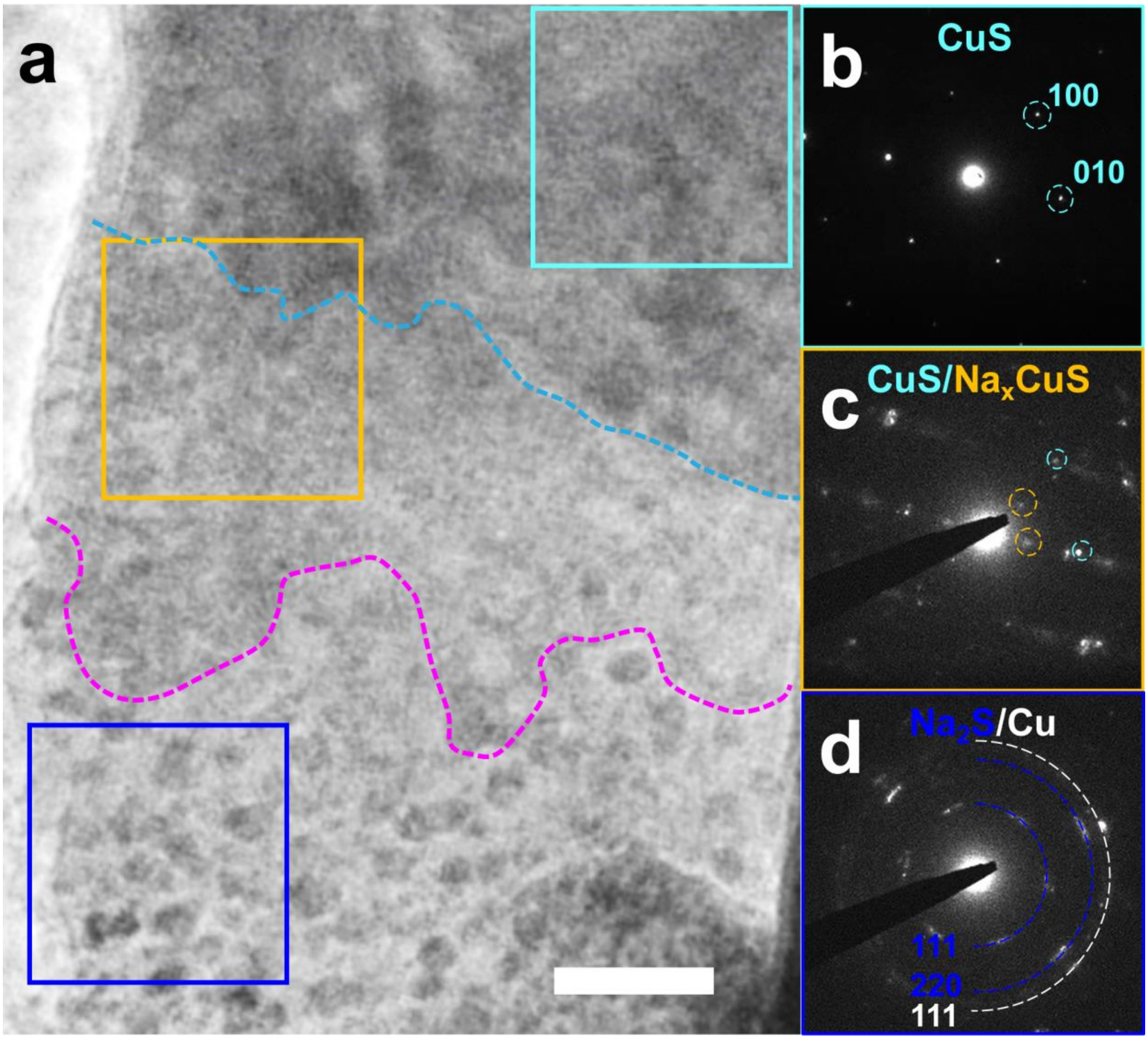

Supplementary Figure 6. Diffraction patterns of pristine, intercalation and conversion reaction regions in the nanoplate upon sodiation. (a) Low magnification TEM image of CuS upon sodiation (scale bar, 20nm). Diffraction patterns of **(b)** CuS, **(c)** CuS/Na$_x$(CuS) and **(d)** Na_2S/Cu, which correspond to cyan, orange and blue-boxed regions in the TEM image. Reflections, marked with orange circles in **(c)**, correspond to {201} planes of $Na_7(Cu_6S_5)_2$.

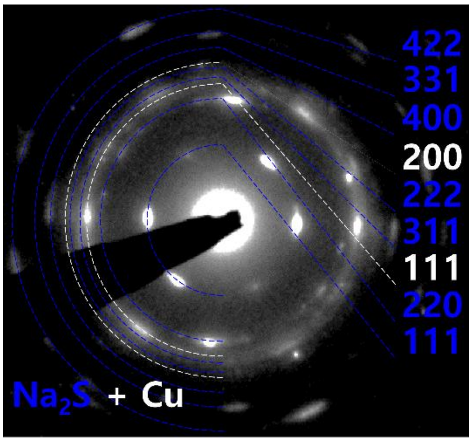

Supplementary Figure 7. **SAED pattern of the fully sodiated CuS nanoplate.**

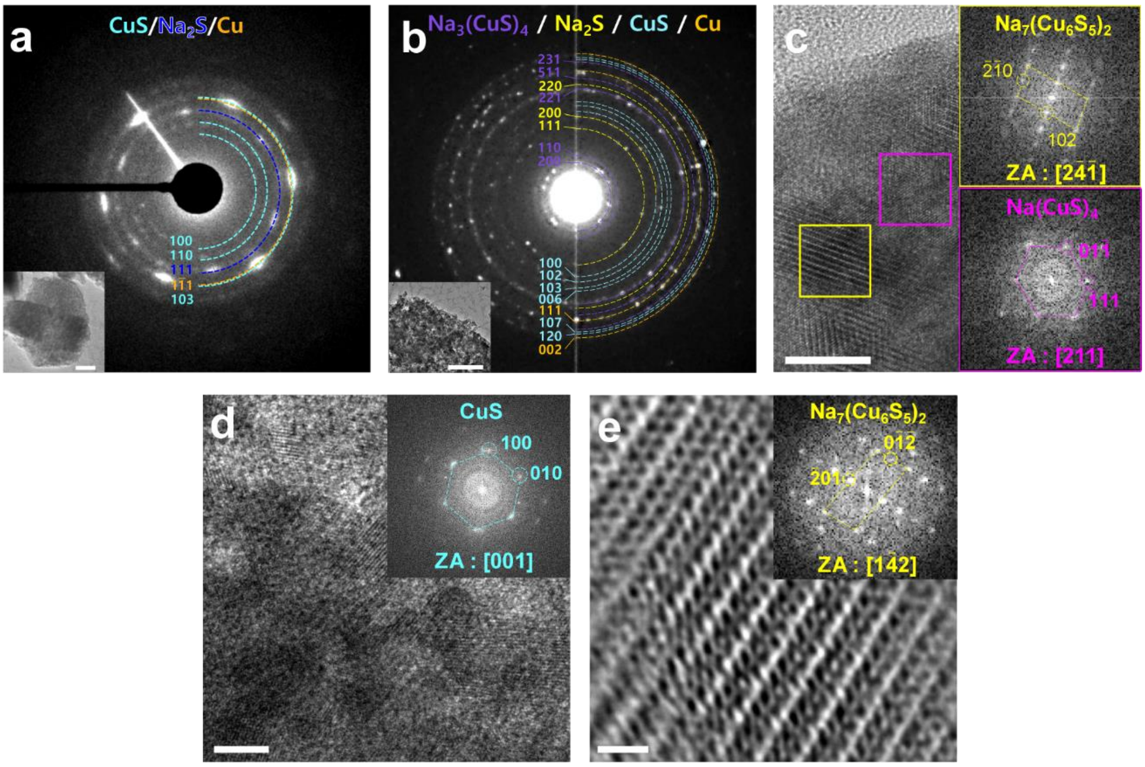

Supplementary Figure 8. Na_xCuS nanoplates observed after 20, 150 and 248 electrochemical cycles. SAED patterns of nanoplates showing the presence of **(a)** CuS, Na_2S and Cu (scale bar, 50 nm) and **(b)** $Na_3(CuS)_4$, Na_2S, CuS and Cu (scale bar, 100 nm). High resolution images of **(c)** $Na(CuS)_4$ and $Na_7(Cu_6S_5)_2$ (scale bar, 10 nm), **(d)** CuS (scale bar, 5 nm), and **(e)** $Na_7(Cu_6S_5)_2$ (scale bar, 1nm) after numerous cycles (20 cycles for **(a)**, **(c)** and **(d)**, 150 cycles for **(b)**, and 248 cycles for **(e)**). An inset figure in **(b)** indicates that a CuS nanoplate is divided into many small pieces after repeated sodiation and desodiation. The ex-situ TEM result confirms that all Na-inserted phases, observed in an in-situ TEM experiment, are formed during the electrochemical cell test. A fully sodiated CuS nanoplate recovers back to CuS upon desodiation. Diffused spots in the fast Fourier transform (FFT) pattern in **(d)** indicate that the nanoplate is fractured into many small crystalline pieces.

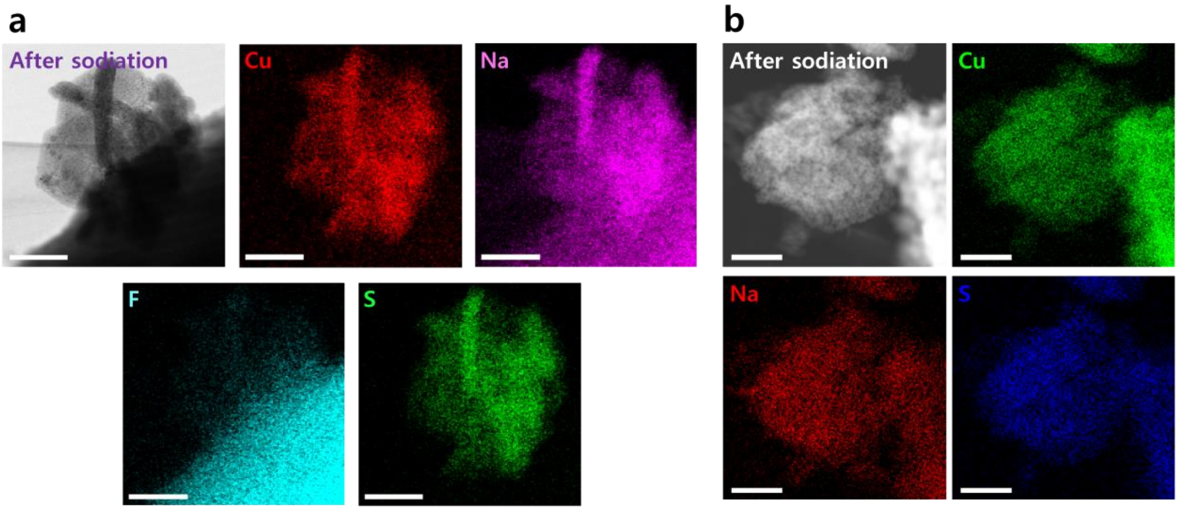

Supplementary Figure 9. Energy dispersive spectroscopy (EDS) elemental mapping of sodiated CuS nanoplates. (a) Bright-field image and the corresponding elemental maps after in-situ TEM sodiation (scale bar, 250 nm). **(b)** Posthumous dark-field image and the corresponding elemental maps after an electrochemical discharge (scale bar, 100nm).

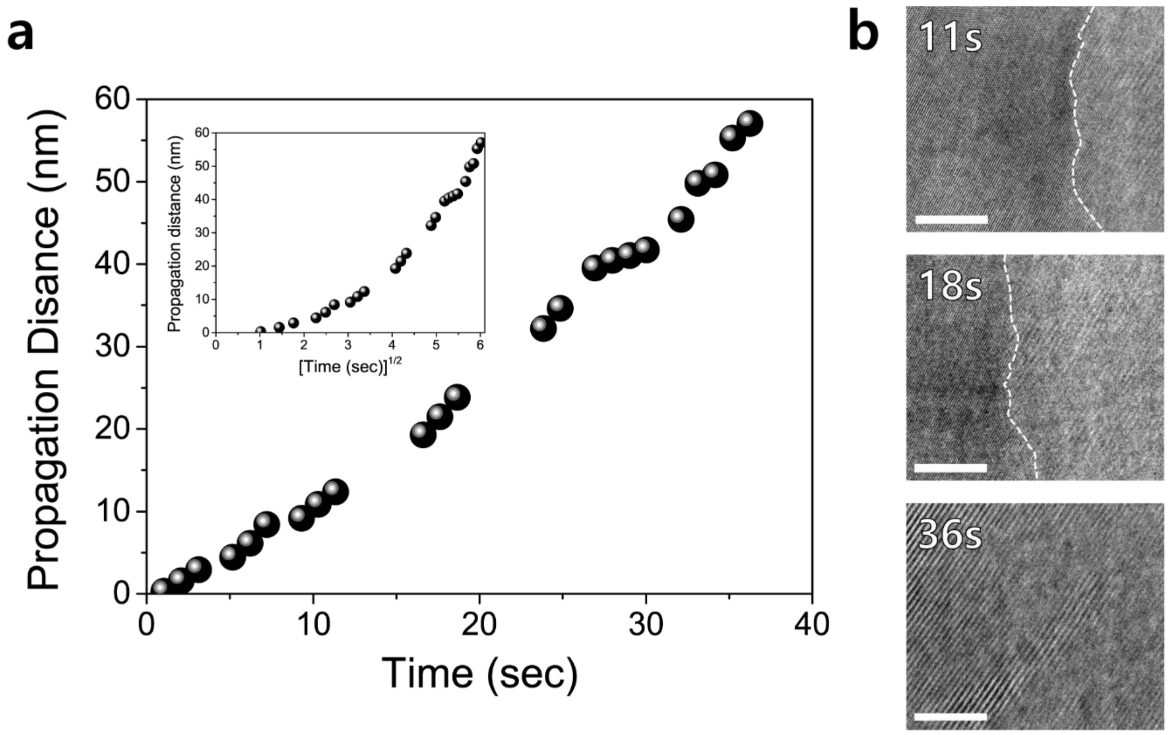

Supplementary Figure 10. Propagation of the intercalation reaction front in CuS. (a) Graph showing propagation distance as a function of electron beam irradiation time. **(b)** Low-magnification TEM images of the reaction front at 11, 18, and 36 s (scale bar, 5nm). Intercalation kinetics is not diffusion-limited but reaction-rate limited since propagation length (L) is not proportional to the square root of time ($\sqrt{t}$) as presented in an inset figure in **(a)**.

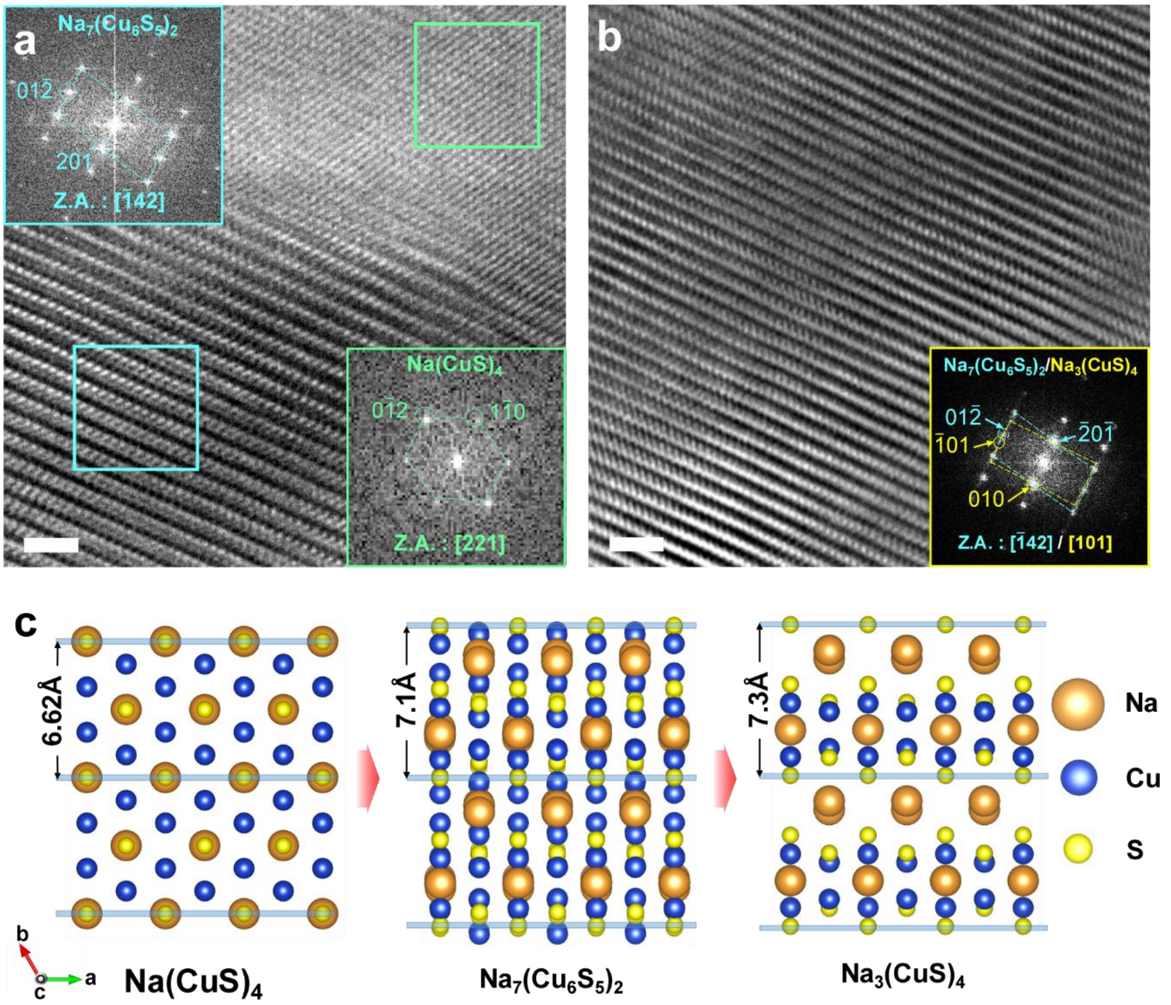

Supplementary Figure 11. HR-TEM observation of phase transitions during the intercalation reaction. Wien-filtered HR-TEM images (scale bar, 2nm) showing **(a)** $Na(CuS)_4$ and $Na_7(Cu_6S_5)_2$ and **(b)** $Na_7(Cu_6S_5)_2$ and $Na_3(CuS)_4$ phases. **(c)** Representative schematics of those phases. Distance between sulfur-containing periodic planes, marked with light blue, increases from 6.62 Å to 7.1 Å and from 7.1 Å to 7.3 Å for $NaCuS_4$ to $Na_7(Cu_6S_5)_2$ and $Na_7(Cu_6S_5)_2$ to Na_3CuS_4 transitions, respectively.

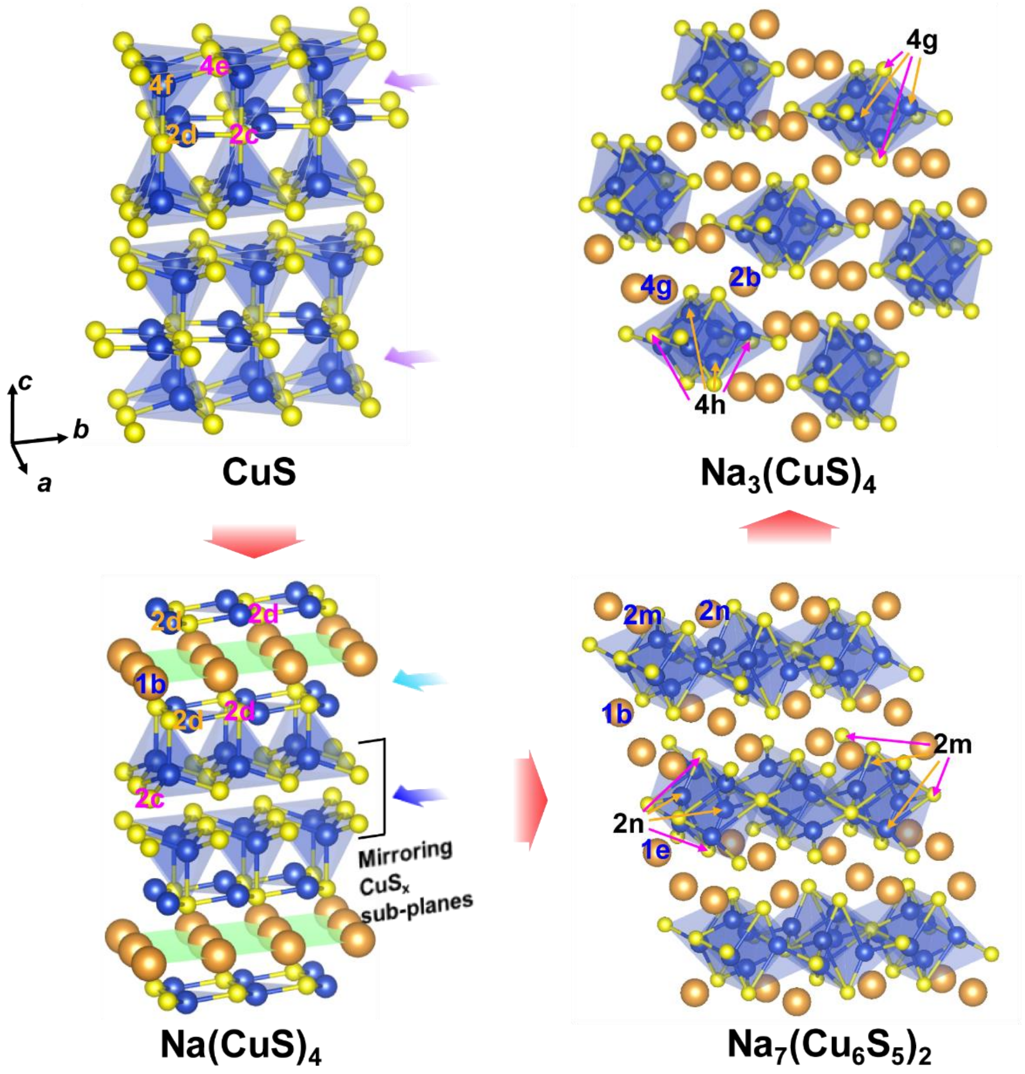

Supplementary Figure 12. **Atomic models of (a) CuS, (b) $Na(CuS)_4$, (c) $Na_7(Cu_6S_5)_2$, and (d) $Na_3(CuS)_4$.** At the first intercalation step, Na atoms are inserted by breaking CuS_x columns and stored between {001} planes to occupy 1*b* sites in a $Na(CuS)_4$ lattice (violet arrow). During this step, Cu atoms, Cu_{4f} and Cu_{2d}, are expelled by 0.12c and 0.02c respectively due to coulombic repulsion. Sulfur atoms are also slightly repositioned. As a result, Cu and occupy 2*d* sites, and S atoms occupy 2*d* and 2*c* sites in a $Na(CuS)_4$ lattice. Lattice parameters along a and b axes increase by 0.62%. With further Na insertion, CuS_x sub-layers experience large separation along [001] by driving more Na atoms into the $Na(CuS)_4$ structure along two paths. First path is the sub-layers where {001} Na planes originally lie (cyan arrow). Second path is the layer formed after the separation of mirroring CuS_x sub-planes (blue arrow). Hence, in the $Na_7(Cu_6S_5)_2$ structure, Na atoms occupy 2*n*, 2*m*, *1b,* and *1e* sites. Half of Cu and S atoms occupy 2*n* sites, while another half occupy 2*m* sites. At the final intercalation step, inserted Na atoms occupy the middle CuS_x columns to divide three CuS_x columns into single columns. As a result, Na atoms occupy 4*g* and 2*b* sites, while Cu and S atoms occupy 4*g* and 4*h* sites in the $Na_3(CuS)_4$ structure.

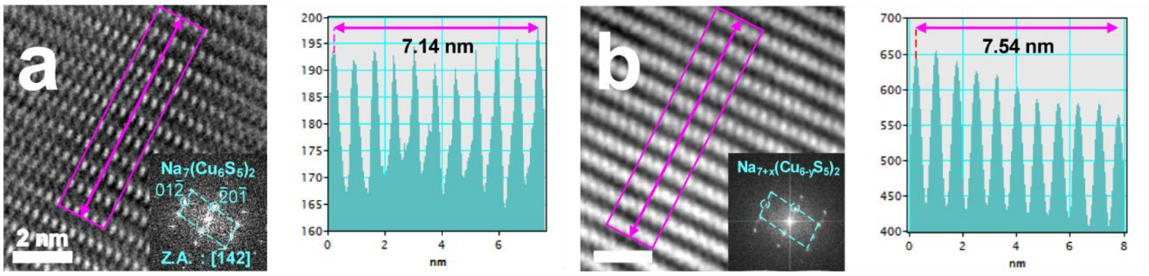

Supplementary Figure 13. Lattice modification of the metastable $Na_7(Cu_6S_5)_2$ phase. (a) Wien-filtered HR-TEM images (scale bar, 2nm) of $Na_7(Cu_6S_5)_2$ phase and **(b)** of modified $Na_7(Cu_6S_5)_2$ phase (speculated to be $Na_{7+x}(Cu_{6-y}S_5)_2$) during sodiation. The images in **(a)** and **(b)** are taken at the same region. We speculate that Na insertion into the $Na_7(Cu_6S_5)_2$ lattice induces a lattice expansion, which generates a peak at ~11.6 degree in XRD (annotated with ▼ in Supplementary Fig. 15).

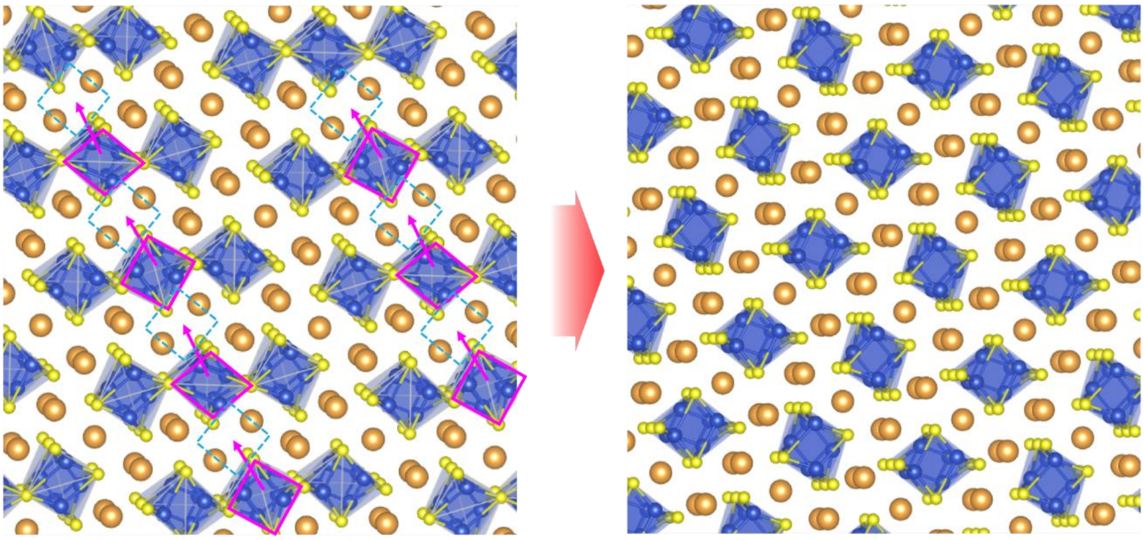

Supplementary Figure 14. Atomic relationship between $Na_7(Cu_6S_5)_2$ and $Na_3(CuS)_4$. $Na_3(CuS)_4$ forms from $Na_7(Cu_6S_5)_2$ by translation of middle CuS_x columns, marked with purple arrows, towards the middle of two sulfur rows, marked with dark cyan-dotted boxes.

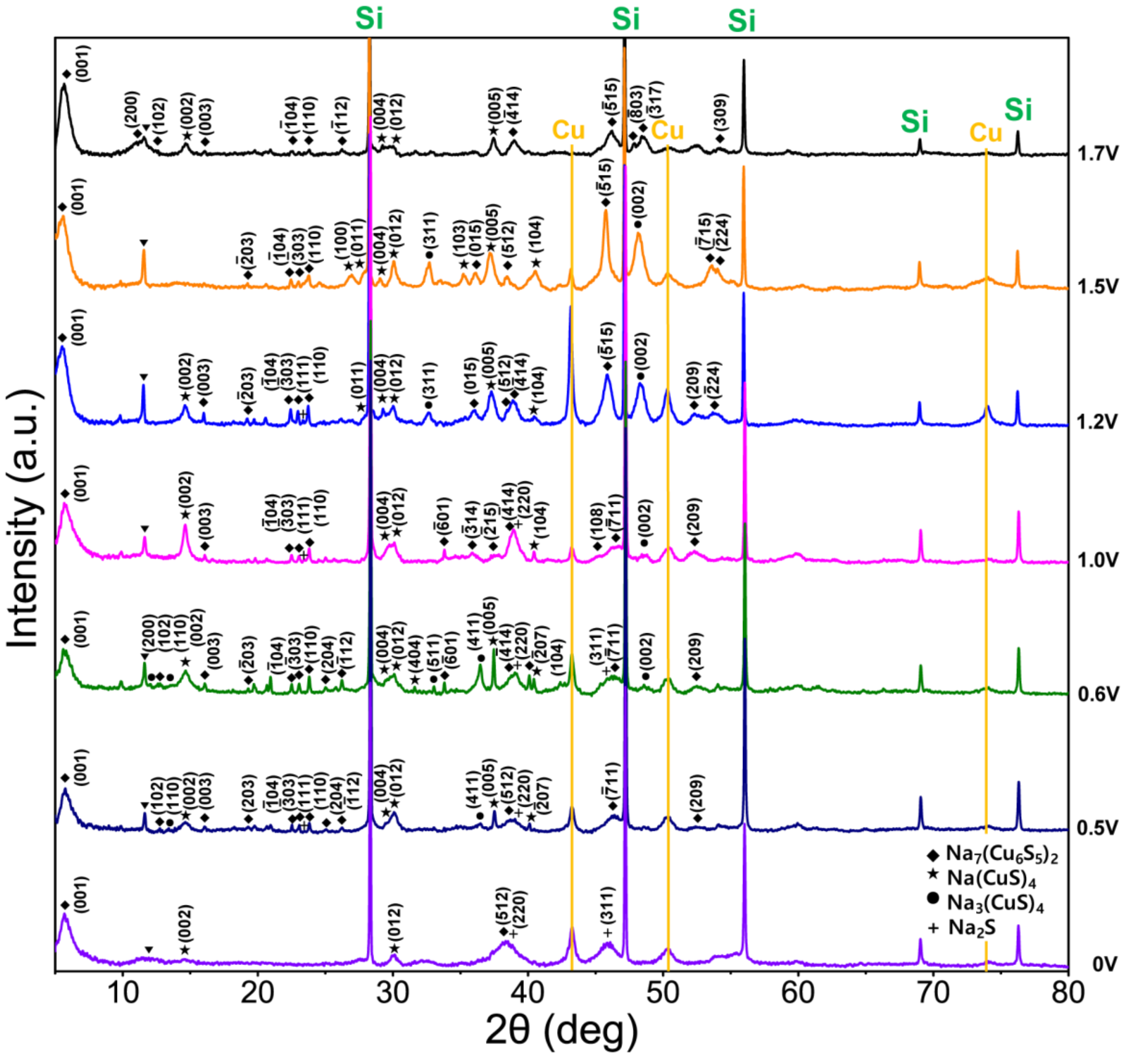

Supplementary Figure 15. Ex-situ x-ray diffraction (XRD) patterns of CuS during discharge. (a) A series of XRD patterns taken at various voltages during discharge process. All phases obtained from in-situ TEM are confirmed with XRD. Both calculated and detected XRD peak information are presented in Table 3. Relative intensity ratio can be different from calculated data (bulk) because of particle size-dependent preferred plane orientation of Na_xCuS nanoparticles[1]. A Cu peak is detected at all voltage levels because of the fragments from a copper electrode foil during XRD sample preparation.

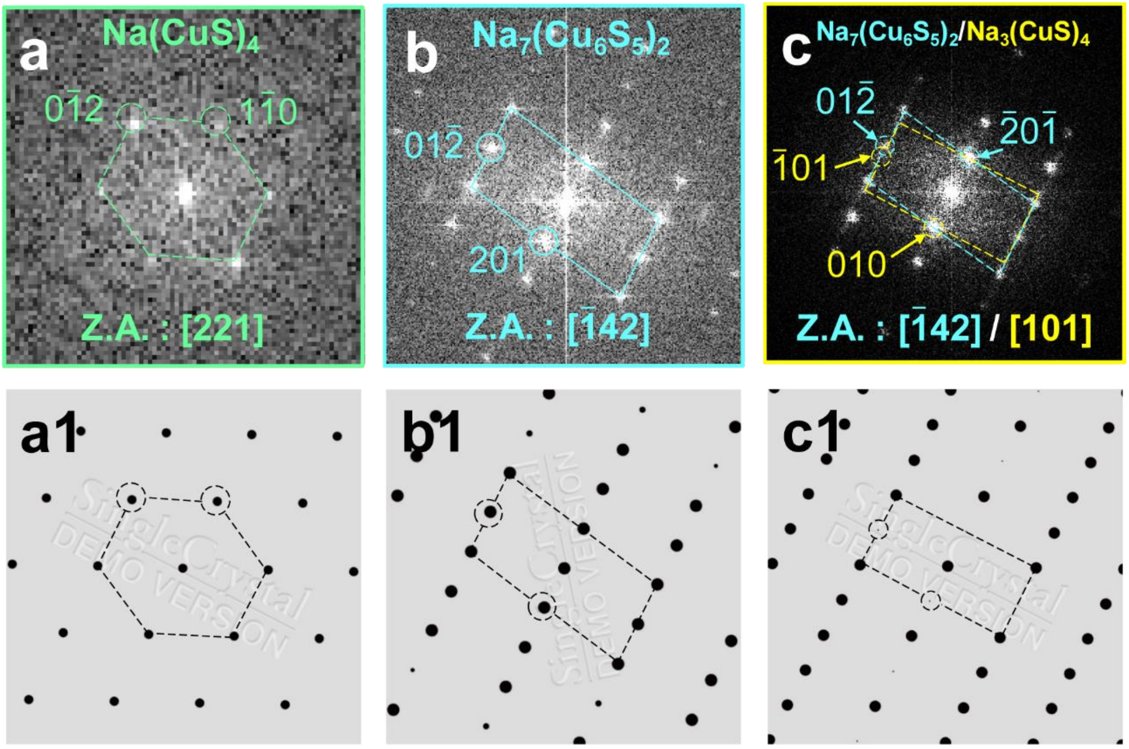

Supplementary Figure 16. Comparison of FFT patterns and simulated diffraction patterns of the intercalation reaction phases. FFT patterns of **(a)** $Na(CuS)_4$, **(b)** $Na_7(Cu_6S_5)_2$ and **(c)** $Na_7(Cu_6S_5)_2$ / $Na_3(CuS)_4$ and simulated diffraction patterns of **(a1)** $Na(CuS)_4$, **(b1)** $Na_7(Cu_6S_5)_2$ and **(c1)** $Na_3(CuS)_4$. Simulated patterns are based on crystallographic information obtained from density function theory (DFT) calculation. There are negligible differences between the data from DFT calculation and inorganic crystal structure database (ICSD), as presented in Table 1 and 2.

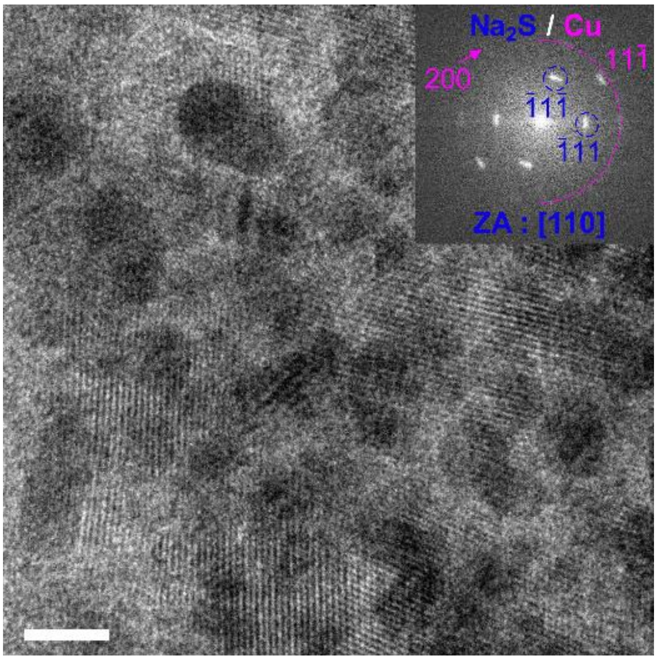

Supplementary Figure 17. HR-TEM image showing Cu nanoparticles embedded in a crystalline Na_2S matrix (scale bar, 5nm).

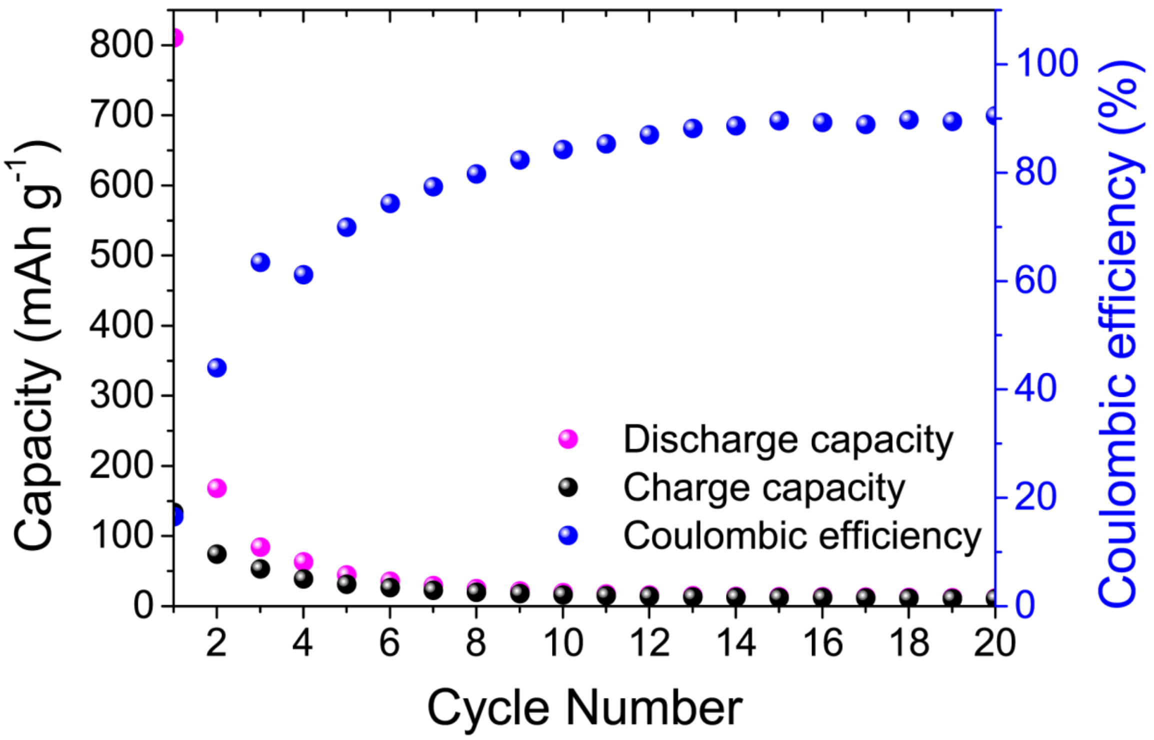

Supplementary Figure 18. Electrochemical performance of CuS nanoplates with a carbonate-based electrolyte. The cell with the carbonate-based electrolyte shows lower coulombic efficiency than the cell with the diglyme-based electrolyte. Furthermore, capacity is not recovered with the carbonate-based electrolyte.

CuS				
2-Theta	d (Å)	Intensity	(hkl)	Detection
27.122	3.285	14	(1,0,0)	O
27.681	3.22	30	(1,0,1)	O
29.277	3.048	65	(1,0,2)	O
31.784	2.813	100	(1,0,3)	O
32.852	2.724	55	(0,0,6)	O
38.835	2.317	10	(1,0,5)	O
47.941	1.896	75	(1,1,0)	O
52.714	1.735	35	(1,0,8)	O
58.681	1.572	16	(2,0,3)	O
59.345	1.556	35	(1,1,6)	O
$Na(CuS)_4$				
2-Theta	d (A)	Intensity	(hkl)	Detection
7.32	12.074	10.3	(0,0,1)	
14.66	6.037	30.4	(0,0,2)	O
26.86	3.317	9.8	(1,0,0)	O
27.87	3.198	12.2	(0,1,1)	O
29.57	3.019	14.8	(0,0,4)	O
30.73	2.907	100	(0,1,2)	O
35.03	2.56	24	(1,0,3)	O
37.2	2.415	14.6	(0,0,5)	O
40.37	2.232	23.9	(1,0,4)	O
46.48	1.952	13.5	(0,1,5)	
47.44	1.915	59.3	(1,1,0)	
53.2	1.72	12.7	(1,0,6)	
57.59	1.599	11.2	(2,0,2)	
$Na_7(Cu_6S_5)_2$				
2-Theta	d (A)	Intensity	(hkl)	Detection
5.447	16.2127	20.3	(0,0,1)	O
5.447	16.2127	20.3	(1,0,0)	O
11.006	8.0324	56.1	(2,0,0)	O
12.12	7.2964	13.4	($\bar{1}$,0,2)	
12.12	7.2964	13.4	($\bar{2}$,0,1)	
12.452	7.1025	100	(1,0,2)	O
12.452	7.1025	100	(2,0,1)	O
15.251	5.8048	12.1	($\bar{2}$,0,2)	
16.389	5.4042	29	(0,0,3)	O
24.379	3.6482	14.6	($\bar{4}$,0,2)	
24.379	3.6482	14.6	(1,1,1)	
32.195	2.7781	11.9	(4,1,0)	
33.716	2.6562	20	($\bar{6}$,0,1)	O
33.716	2.6562	20	($\bar{2}$,1,4)	O
34.942	2.5657	13.1	($\bar{5}$,0,4)	
34.942	2.5657	13.1	($\bar{4}$,0,5)	
35.971	2.4947	5.6	($\bar{3}$,1,4)	O
36.202	2.4793	27.3	(0,1,5)	O
36.202	2.4793	27.3	(5,0,4)	O
36.687	2.4476	20.5	(4,1,3)	
36.687	2.4476	20.5	($\bar{5}$,1,1)	
36.984	2.4286	12.6	(5,1,1)	
36.984	2.4286	12.6	(6,0,3)	
38.398	2.3424	28.6	(5,1,2)	O

38.836	2.317	10.3	$(\bar{4},1,4)$	O
38.836	2.317	10.3	(0,0,7)	O
41.015	2.1988	20.1	$(\bar{1},1,6)$	
41.015	2.1988	20.1	(6,1,0)	
46.225	1.9624	19.5	$(\bar{7},1,1)$	O
46.225	1.9624	19.5	(1,1,7)	O
46.821	1.9387	17.3	$(\bar{2},1,7)$	
46.821	1.9387	17.3	(5,1,5)	
47.215	1.9235	32.1	(0,2,0)	
$Na_3(CuS)_4$				
2-Theta	d (A)	Intensity	(hkl)	Detection
12.1	7.31	59	(2,0,0)	O
13.76	6.43	100	(1,1,0)	O
24.34	3.654	11	(4,0,0)	
32.5	2.753	11	(3,1,1)	O
33.06	2.707	19	(5,1,0)	
36.47	2.462	18	(4,1,1)	O
36.71	2.446	33	(2,2,1)	
39.28	2.292	28	(3,2,1)	
46.69	1.944	21	(2,3,1)	
48.21	1.886	16	(0,0,2)	O
51.38	1.777	11	(6,2,1)	
Na_2S				
2-Theta	d (A)	Intensity	(hkl)	Detection
23.52	3.78	57.6	(1,1,1)	O
27.22	3.273	4.5	(2,0,0)	
38.88	2.315	100	(2,2,0)	O
45.94	1.974	17.5	(3,1,1)	O
Cu				
2-Theta	d (A)	Intensity	(hkl)	Detection
43.2	2.093	100	(1,1,1)	O
50.31	1.812	43	(2,0,0)	O
73.9	1.282	17.7	(2,2,0)	O

Supplementary Table 1. Major peaks from Calculated XRD information of CuS, $Na(CuS)_4$, $Na_7(Cu_6S_5)_2$, $Na_3(CuS)_4$, Na_2S and Cu based on ICSD.

$Na(CuS)_4$<br>Space group / crystal system : $P\bar{3}m1$ [164] / trigonal<br>Lattice parameter (a, b, c / α, β, γ) : 3.820Å, 3.820Å, 12.156Å / 90.000°, 90.000°, 120.000°			
Atom	x	y	z
Na	0.0000	0.0000	0.5000
Cu(1)	0.6667	0.3333	0.3115
Cu(2)	0.3333	0.6667	0.1484
S(1)	0.3333	0.6667	0.3409
S(2)	0.0000	0.0000	0.0868
$Na_7(Cu_6S_5)_2$<br>Space group / crystal system : *P2/m* [10] / monoclinic<br>Lattice parameter (a, b, c / α, β, γ) : 3.787Å, 16.162Å, 16.229Å / 90.374°, 90.000°, 90.000°			
Atom	x	y	z
Na(1)	0.5000	0.5000	0.0000
Na(2)	0.0000	0.3153	0.1042
Na(3)	0.0000	0.1190	0.7091
Na(4)	0.5000	0.2806	0.5935
Na(5)	0.5000	0.0000	0.0000
Na(6)	0.0000	0.1976	0.9150
Na(7)	0.0000	0.3940	0.3040
Na(8)	0.5000	0.2025	0.3934
Cu(1)	0.5000	0.5088	0.4197
Cu(2)	0.0000	0.5880	0.4986
Cu(3)	0.5000	0.5527	0.2671
Cu(4)	0.0000	0.6763	0.2480
Cu(5)	0.5000	0.6508	0.1343
Cu(6)	0.0000	0.5237	0.1529
Cu(7)	0.5000	0.0744	0.5509
Cu(8)	0.0000	0.9537	0.5658
Cu(9)	0.5000	0.9867	0.8034
Cu(10)	0.0000	0.9127	0.7222
Cu(11)	0.5000	0.8128	0.8058
Cu(12)	0.0000	0.8885	0.8925
S(1)	0.0000	0.5778	0.3542
S(2)	0.5000	0.3697	0.4380
S(3)	0.5000	0.4438	0.1754
S(4)	0.5000	0.7568	0.2324
S(5)	0.0000	0.6265	0.0581
S(6)	0.5000	0.9778	0.6558
S(7)	0.0000	0.1534	0.5285
S(8)	0.0000	0.0293	0.8644
S(9)	0.0000	0.7735	0.7372
S(10)	0.5000	0.1745	0.0532
$Na_3(CuS)_4$<br>Space group / crystal system : *Pbam* [55] / orthorhombic<br>Lattice parameter (a, b, c / α, β, γ) : 3.741Å,7.244Å, 14.558Å / 90.374°, 90.000°, 90.000°			
Atom	x	y	z
Na(1)	0.5000	0.5000	0.5000
Na(2)	0.0000	0.5202	0.2640
Cu(1)	0.5000	0.4215	0.9111
Cu(2)	0.0000	0.3321	0.0610
S(1)	0.5000	0.2855	0.1511
S(2)	0.0000	0.2546	0.4086

Supplementary Table 2. Crystallographic information of $Na(CuS)_4$, $Na_7(Cu_6S_5)_2$, and $Na_3(CuS)_4$ phases based on DFT calculation[2].

Na(CuS)₄			

$Na(CuS)_4$<br>Space group / crystal system : $P\bar{3}m1$ [164] / trigonal<br>Lattice parameter (a, b, c / α, β, γ) : 3.830Å, 3.830Å, 12.074Å / 90.000°, 90.000°, 120.000°			
Atom	x	y	z
Na	0.000	0.000	0.500
Cu(1)	0.6667	0.3333	0.3108
Cu(2)	0.3333	0.6667	0.1477
S(1)	0.3333	0.6667	0.3414
S(2)	0.000	0.000	0.0863
$Na_7(Cu_6S_5)_2$<br>Space group / crystal system : *P2/m* [10] / monoclinic<br>Lattice parameter (a, b, c / α, β, γ) : 16.074Å, 3.847Å, 16.222Å / 91.940°, 90.000°, 90.000°			
Atom	x	y	z
Na(1)	0.500	0.500	0.000
Na(2)	0.3184	0.000	0.1006
Na(3)	0.1193	0.000	0.7062
Na(4)	0.2783	0.500	0.5964
Na(5)	0.000	0.500	0.000
Na(6)	0.1943	0.000	0.9134
Na(7)	0.3931	0.000	0.3041
Na(8)	0.2044	0.500	0.3932
Cu(1)	0.5153	0.500	0.4216
Cu(2)	0.5986	0.000	0.4948
Cu(3)	0.5516	0.500	0.2679
Cu(4)	0.6817	0.000	0.2445
Cu(5)	0.6537	0.500	0.1325
Cu(6)	0.5184	0.000	0.1518
Cu(7)	0.0783	0.500	0.5514
Cu(8)	-0.0465	0.000	0.5692
Cu(9)	-0.0016	0.500	0.7993
Cu(10)	-0.0832	0.000	0.7293
Cu(11)	-0.1925	0.500	0.8081
Cu(12)	-0.1196	0.000	0.8971
S(1)	0.5830	0.000	0.3497
S(2)	0.3758	0.500	0.4389
S(3)	0.4413	0.500	0.1750
S(4)	0.7616	0.500	0.2314
S(5)	0.6237	0.000	0.0621
S(6)	-0.0194	0.500	0.6536
S(7)	0.1539	0.000	0.5274
S(8)	0.0213	0.000	0.8644
S(9)	-0.2231	0.000	0.7382
S(10)	0.1786	0.500	0.0500
$Na_3(CuS)_4$<br>Space group / crystal system : *Pbam* [55] / orthorhombic<br>Lattice parameter (a, b, c / α, β, γ) : 14.624Å, 7.163Å, 3.771Å / 90.000°, 90.000°, 90.000°			
Atom	x	y	z
Na(1)	0.2666	0.5155	0
Na(2)	0.5	0.5	0.5
Cu(1)	0.4067	0.0795	0.5
Cu(2)	0.0647	0.3321	0
S(1)	0.1532	0.2834	0.5
S(2)	0.4119	0.2514	0

Supplementary Table 3. Crystallographic information of $Na(CuS)_4$, $Na_7(Cu_6S_5)_2$, and $Na_3(CuS)_4$ phases based on ICSD[3,4,5].

Supplementary References

1. Peck, M. A. & Langell, M. A. Comparison of Nanoscaled and Bulk NiO Structural and Environmental Characteristics by XRD, XAFS, and XPS. *Chem. Mater.* **24**, 4483–4490 (2012).

2. Jain, A., et al. Commentary: The Materials Project: A materials genome approach to accelerating materials innovation. *APL Mater.* **1**, 011002 (2013).

3. Zhang, X., Kanatzidis, M. G., Hogan, T. & Kannewurf, C. R. $NaCu_4S_4$, a Simple New Low-Dimensional, Metallic Copper Polychalcogenide, Structurally Related to CuS. *J. Am. Chem. Soc.* **118**, 693–694 (1996).

4. Klepp, K. O., Sing, M. & Boller, H. Preparation and crystal structure of $Na_7Cu_{12}S_{10}$, a mixed valent thiocuprate with a pseudo-one-dimensional structure. *J. Alloy. Compd.* **198**, 25–30 (1993).

5. Burschka, C., $Na_3Cu_4S_4$-ein Thiocuprat mit unverknüpften 1∞[Cu_4S_4] -Ketten / $Na_3Cu_4S_4$-a Thiocuprate with Isolated 1∞[Cu_4S_4]-Chains. In *Zeitschrift für Naturforschung B* **34**, 396 (1979).
