# Supplementary material for: Atomic visualization of a non-equilibrium sodiation pathway in copper sulfide
Source: Nat Commun. 2018 Mar 2;9:922. doi: 10.1038/s41467-018-03322-9 (PMC5834500; doi:10.1038/s41467-018-03322-9)
Supplement: Supplementary file 3 — Description of Additional Supplementary Files [file 41467_2018_3322_MOESM3_ESM.pdf]

Description of Additional Supplementary Files

File Name: Supplementary Movie 1
Description: **Sodiation process of a CuS nanoplate**. Low-magnification transmission electron microscopy (TEM) movie showing an overlapped intercalation-conversion reaction process. Na insertion induces the intercalation reaction, indicated by propagation of the first front. After 24 s, the second front, indicative of conversion reaction, also emerges. This suggests that the conversion reaction happens even before the end of intercalation reaction. The second front catches up the first front at 163 s, and they eventually become indistinguishable from each other. The movie plays 8 times faster.

File Name: Supplementary Movie 2
Description: **Sodiation process of a CuS nanoplate at high resolution**. High-resolution TEM (HR-TEM) movie showing that the intercalation reaction happens along the <210> direction, followed by the conversion reaction. The movie plays 3 times faster.
